# The Etiology of Moebius Syndrome—Making the Case for Animal Models

**DOI:** 10.3390/ijms26094217

**Published:** 2025-04-29

**Authors:** Manuela-Petronela Tracicaru, Rareș-Vasile Tracicaru, Delia Hînganu, Marius Valeriu Hînganu

**Affiliations:** Department of Morphofunctional Sciences, Anatomy and Embryology, Grigore T. Popa University of Medicine and Pharmacy Iași, University Street No 16, 700115 Iași, Romania; manuela-petronela.tracicaru@umfiasi.ro (M.-P.T.); hinganu.delia@umfiasi.ro (D.H.); marius.hinganu@umfiasi.ro (M.V.H.)

**Keywords:** Moebius syndrome, Moebius sequence, animal model, mice, PLXND1, REV3L, TUBB3

## Abstract

Moebius syndrome (MBS) is a rare disease consisting of uni-/bilateral palsy of CN VI and VII without impairment of vertical eye movements. Its uncommon nature means that the etiology is still uncertain. It is thought to be caused by vascular lesions leading to infarction in the nuclei of cranial nerves VI and VII on the posterior aspect of the pons. However, several genes have also been discussed as possibly causative. We performed a literature search in the PUBMED database and on the Science Direct platform with terms related to the pathology and to each etiology individually. Included were original papers and review articles published in peer-reviewed international journals and reference books and databases on the subjects discussed. We excluded articles not published in English, conference communications, dissertations, monographs, and other non-peer-reviewed forms of publication. The total number of publications thus included was 62. This review discusses the functions of the three most related genes found in recent research (PLXND1, REV3L, TUBB3) and the results of animal studies focusing on their mutations. We note that the PLXND1 and REV3L mutations have been most associated with MBS and that the current studies on their function suggest histological lesions similar to the target disease, albeit without clear phenotypic expression. We ascertain that TUBB3 mutations are mostly related to CEFOM3, which is a differential diagnosis for MBS. Regarding the vascular etiology, we review the types of lesions involved and discuss their timing in relation to embryologic stages. We also highlight the main investigation methods available. A multitude of the factors discussed might be causative of MBS, and we thus consider it necessary to attempt the development of an animal model for the disease. To this end, we propose the development of transgenic mice models containing the single nucleotide mutations documented in human patients, and we discuss the use of the chick embryo model for the vascular etiology.

## 1. Introduction

Moebius syndrome or sequence (MBS) is a congenital, non-progressive palsy of the 6th and 7th pair of cranial nerves that can be either unilateral or bilateral. Minimum diagnostic criteria were established in 2007 and are as follows: “congenital, non-progressive facial weakness with limited abduction of one or both eyes and full vertical motility” [1,2,3,4]. It is thus very important to distinguish the syndrome from other cranial nerve palsies such as congenital fibrosis of the extraocular muscles (CFEOM), which presents with vertical gaze palsy [5], or syndromes that involve multiple cranial nerve palsies and reside under the umbrella of brainstem dysgenesis [6]. Although these features were established, there are still many cases that do present abnormalities of vertical gaze or associated palsies and are classified as MBS [1,5], falling in the category of atypical MBS. Associations with the Poland syndrome (unilateral hypoplasia of the pectoralis muscle associated with syndactyly or other upper limb and chest abnormalities) have also been widely reported in the literature. This would favor a vascular origin, since the Poland syndrome is caused by subclavian artery malformations, which could also affect the vertebral artery (a branch of the subclavian artery) and, thus, the blood flow to the brainstem [7,8,9]. The rarity of the disease and the difficulty of diagnosis mean that the real incidence is unknown. Reports from Italy present an incidence of 0.06/10,000 births [3], while the same authors cite a prevalence of 0.002% in a Dutch series [3]. A review from 2019 found 395 reported cases of MBS [1]; however, when considering the criterion of normal vertical gaze, the number of classical MBS cases would be lower. Furthermore, since so few cases are published and the reporting varies wildly, it is very difficult to ascertain a unified theory about the etiology of this disease.

A number of theories have been discussed. The first to arise, and still one of the most plausible, is the vascular theory, by which infarction within the area of the CN VI and VII nuclei causes the deficit [2,4,10]. This is supported by the aspect of necrosis within these nuclei encountered histologically and on imaging studies [2,4,10,11,12,13]. The involvement of teratogens also tie back to the same theory as cocaine, ethanol, thalidomide, misoprostol, and ergotamine consumption, which have all been linked to MBS [2,12] and affect the microvascularization of the brainstem through induced hypoxia [10]. The second main theory discussed in relation to MBS is the genetic disruption of two key genes, PLXND1 and REV3L, presented in a recent series of cases [7,14,15], with this being still a disputed subject [16]. Another possibly implicated gene is considered to be TUBB3 [5], which is known to be involved in the etiology of CFEOM 3 [5,17,18], an important differential diagnosis for MBS.

It is the authors’ opinion that accurate investigation of these possible causes cannot be performed solely on patients suffering from the disease because of the rarity of MBS and because the changes occur during the congenital period. Thus, an animal model of MBS would be the ideal method of investigation. This review explores the characteristics of each gene and their functions and highlights previous animal studies involving the target genetic defects. Furthermore, we discuss the utility of animal models in elucidating the mechanisms of vascular injury in MBS. Our ultimate aim is to develop an animal model that permits further studies of this pathology and elucidates the exact pathogenic sequence that leads to it.

## 2. Results

### 2.1. The Role of PLXND1 in Disease Pathogenesis in Human and Animal Models

**Figure 1:**
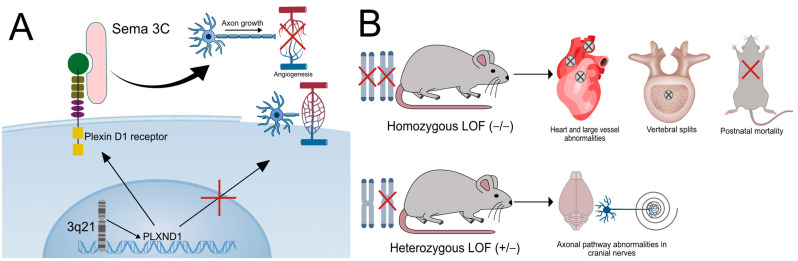
PLXND1 function: Image (**A**) details the role of the PLXND1 gene. Located on chromosome 3q21, it codes a transmembrane protein (Plexin D1) involved in semaphorin signaling and found on endothelial neovascular cells. Interaction with semaphorin 3C (Sema 3C) signals from developing neurons halts incursions of new blood vessels in the path of the developing axon. A lack of Plexin D1 leads to aberrant vasculogenesis and a disruption of normal axonal growth. Image (**B**) details the results of mice experiments into Plexin D1 function. Homozygous loss-of-function (LOF) mutations cause cardiac and large vessel abnormalities, leading to rapid postnatal lethality. Vertebral splits have also been documented. Heterozygous LOF mutations lead to abnormal axonal pathfinding, especially involving the cranial nerves.

PLXND1 (ID: 2319) is a gene situated on chromosome 3q22.1 (NC_000003.12, ref. GRCh38.p14) that codes the Plexin D1 protein [19], from the larger group of plexins, which are receptors for semaphorins, a group of proteins involved in axonal pathway generation (Figure 1A) [20]. It is present in all vertebrates and has wide tissue distribution, with maximum representation being found in placenta and fat tissues. In the brain, its expression is moderate (RPKM = 8.598, sd of 1.751) [19]. Its role in central nervous system (CNS) development is well established, as it appears alongside its ligand, Sema 3E, during synaptic differentiation [21]. Semaphorins in general are considered to be chemorepellents for neurons [20]. However, at least in the case of Plexin D1-mediated chemotaxis, its function promotes an increase in synaptic density in the neocortical areas evaluated [21]. The authors propose that this is not contradictory to the chemorepellent role, the increase in synaptic density being attributed to improved efficiency because of specific synaptic guidance [21,22]. This was supported by a recent study that demonstrated the necessity of Plexin D1 as a receptor on neovascular endothelial cells. Endothelial cells expressing Plexin D1 are essential to the adequate distribution of ventral root motor neurons, since the receptor receives Sema 3C signaling from developing axons. These signals prevent neovasculogenic development in the path of the spinal nerve (Figure 1A) [23]. Embryos possessing mutant or null (knockdown/knockout) Plexin D1 showed aberrant vasculogenesis, with vascular structures encroaching on motor neuron development and preventing normal pathfinding in both mice and chickens (Figure 1A) [23]. Sensitive neurons seem not to control vascular growth through the same mechanism, although a lack of Plexin D1 in endothelial cells cultured alongside sensory neurons led to a decrease in axonal development in general, with neural growth hindered by the endothelial barrier [23]. This behavior seems to be mediated only by Sema 3C signaling, while knockout variants for Sema 3E and 4A did not exhibit affected patterning at the vascular/neuron barrier [23]. However, other studies have remarked on the importance of Sema 3E signaling in vasculogenesis within the nervous system as a whole [24]. The role of Plexin D1 in endothelial cells goes beyond vasculogenesis, as it has been shown to be an independent factor in dictating the response of arterial walls to mechanical stress in mice, its absence determining a decrease in protective inflammatory signaling and poor organization of stress-resisting actin filaments [25].

Plexin D1 involvement in embryogenesis has been documented in animal models. Thus, knockout homozygous (−/−) mice embryos exhibit multiple cardiac and large-vessel abnormalities consisting of persistent arterial trunk along with outflow tract malformations and coronary artery malformations [26]. This leads to death shortly after birth [26]. Peripheral vascular defects are also seen, although with less phenotypic impact (Figure 1B) [26]. All these defects are, again, linked to Sema 3C binding [27]. Skeletal abnormalities have also been observed in homozygous knockout mice, where vertebral splits and abnormal costal implantation were described (Figure 1B) [27,28]. This was attributed to invasion of the bone by abnormal microvascular structures, reminiscent of the mechanism discussed regarding neural pathfinding abnormalities [28]. Postnatal loss of function reinforced the idea that Plexin D1 is critical for vascular structure, as defective mice showed retinal angiopathy [27]. When looking at congenital neuron defects, PLXND1 has been reported in association with classical MBS in a cohort of 12 patients [14], with the same study documenting that in both knockdown (+/−) and knockout (−/−) mice, a subpopulation of motor neurons within the primordial facial nerve area (r4/r5) showed impaired migration (Figure 1B). This was similar to patients exhibiting the c.5685C>A; p.Asn1895Lys; NM_015103, c.4454_4455GC4CA; p.Arg1485Pro and c.3018C4T; p.Leu1006Leu mutations of the PLXND1 gene [14]. A mutation within PLXND1 (c.2890G>A p.V964M) has been reported in a case of Poland–Moebius syndrome, a well-known association of classical MBS [7]. A more recent study examined a cohort of 37 patients with MBS using a clinical-grade exome test and identified mutations in the PLXND1 gene in 8 of the patients, without de novo origins, all being inherited forms from clinically healthy parents [16]. Considering that the previous study did not identify mutations in the gene in most patients [14], we can assume that PLXND1 plays a role in the pathogenesis of Moebius but is not necessary for its existence. Incomplete penetrance may be also involved in the varying phenotypic expressions for mutated genes [16]. PLXND1 mutation, however, would be a perfect candidate for the etiology of MBS, since it would explain both the neural disruptions and the hindbrain involution due to vascular lesions that are observed in MBS.

### 2.2. The Role of REV3L in Disease Pathogenesis in Human and Animal Models

**Figure 2:**
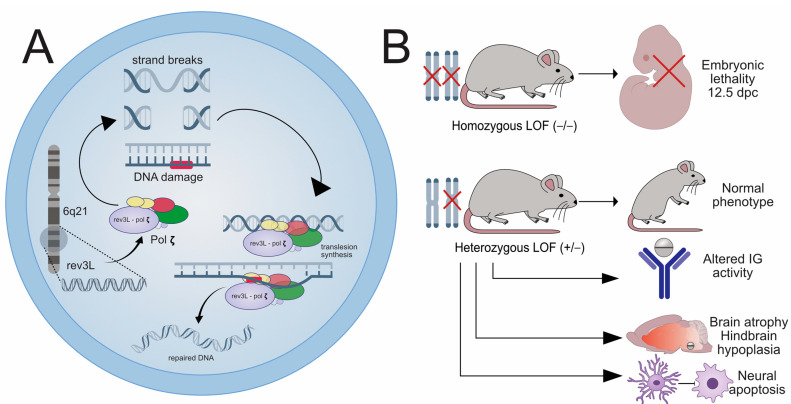
REV3L function: Image (**A**) details the role of the REV3L gene. Located on chromosome 6q21, it codes the catalytic (ζ) subunit of the DNA polymerase (pol ζ). This polymerase is active in repairing single- or double-strand breaks and in performing translesion synthesis repair of damaged DNA. Image (**B**) details the results of mice experiments into REV3L function. Homozygous loss of function (LOF) leads to intrauterine lethality at 12.5 dpc. Heterozygous LOF mutations lead to mice presenting normal phenotypes and preserved fertility. However, these mice present altered immune responses due to affected immunoglobulin activity, brain atrophy and hindbrain hypoplasia, and signs of neural apoptosis with decreased concentrations of neurons within the facial (CN VII) nucleus.

REV3L (ID: 5980) is a gene located on chromosome 6q21 (NC_000006.12, GRCh38.p14) that codes the assembly of the catalytic zeta (ζ) subunit of DNA polymerase (pol ζ) [19]. Pol ζ acts as a repair polymerase for acquired DNA damage, especially in the forms of strand breaks [29]. Pol ζ is also involved in translesion synthesis, a mutation tolerance process that is prone to fidelity-related errors (Figure 2A) [29,30]. It is found in all eukaryotes, albeit with interspecies differences [30]. In mammals, pol ζ is crucial, as all forms of DNA damage are repaired in the presence of this polymerase [29]. Its absence determines the accumulation of strand brakes and other mutations within a couple of cell cycles, usually leading to apoptosis [30]. Their function is, however, limited by low processive speeds; since an overexpression could lead to rapid accumulation of mutations within cells, evolutionary pressure limited the impact of translesion polymerases such as pol ζ [30]. The clinical impact of pol ζ/REV3L inactivation has been studied in several animal models [29,31,32,33]. Yeast (*S. cerevisae*) expresses pol ζ through two complementary catalytic domains, Rev3p/Rev7p, and is a useful model for testing REV3L mutagenesis [30]. Further studies have focused on the involvement of REV3L in human disease [14,15,34]. The presence of REV3L is essential for embryonic development, since knockout mice (REV3L −/−) models show intrauterine lethality [29,30,31,33]. This seems to happen in mice starting from 7.5 dpc, with mutagenesis-induced malformations accumulating over the following days (Figure 2B) [31]. This affects mostly the mesoderm during this period of embryogenesis, resulting in heart development abnormalities [31] with lethality by 12.5 dpc. Another author reported even quicker lethality for some homozygous mutations of REV3L [29]. In mice where knockout was performed after the embryonic period, an increase in the frequency and lethality of acquired cancers was observed, especially in p53-deficient mice [29]. Similar experiments on immortalized human cell lines with REV3L knockout revealed the upregulation of the interferon-related immune response through the cGAS-STING pathway and, consequently, greatly accelerated cell senescence [32]. That this effect is triggered by the absence of pol ζ was also certified by the authors by reversing the inflammatory effect when introducing an active replacement construct in the knockout cells [32]. Heterozygous deletion of the REV3L did not seem to affect the development of the mice significantly, with populations of heterozygous mice keeping up in size and number with those of wildtype mice and greatly outgrowing the homozygous ones (Figure 2B) [31]. The results were similar on immortalized human cell lines, where REV3L +/− cells retained pol ζ activity and showed similar immune responses to wildtype cells [32]. When catalytic subunit inactivation mice were used (ATA), homozygous mice (ATA/ATA) showed embryonic lethality as previously described, and heterozygous mice (ATA/wt) seemed to have normal development and fertility [33]. However, other authors reported a decrease in immune specificity for heterozygous mice [29]. Other non-lethal mutations of the catalytic subunit were induced, and altered immunoglobulin activity was observed [33]. All these studies suggest that REV3L has a complex role in the development of embryos and is involved in numerous mutagenesis-related processes postpartum.

Regarding its role in the development of MBS, one group identified multiple SNC mutations (c.1096+1G>A; c.1160A>G; c.2662A>T) in three patients with MBS, leading to decreased transcription of pol ζ for the first case and loss of function for the others, respectively. Heterozygous mice exhibiting the mutations presented with hindbrain hypoplasia and brain atrophy (with a compensatory enlargement of the subarachnoid area). Furthermore, a decrease in density in the motor neurons within the facial nucleus was observed. REV3L homozygous (−/−) mice presented again with embryonic lethality and brain-wide apoptosis. DNA damage due to the mutations was widespread, contributing to further destruction of the neurons in both mouse variants (−/− and +/−) [14]. In a subsequent cohort study, only one mutation in the REV3L gene was identified (c.3153G>T), which was considered to be a missense mutation and deemed to be not causative [16] for MBS. REV3L has also been involved in the etiology of other pathologies. A case of a homozygous REV3L mutation (c.8258C>G) was reported for a boy presenting with cranio-facial deformities, skeletal abnormalities, and impaired cognitive development. An increase in the expression of pigmentated nevi was also noted, both in the patient and in the heterozygous members of the family. What is also interesting is that poor facial movement and convergent strabismus were also noted (suggesting palsies of the 6th and 7th cranial nerves) but no diagnosis of MBS was noted [15]. Pol ζ deletion was also found to play a significant mutagenic role in 15 cancer types recently analyzed by 1 group [34]. The clinical impact was positive in these patients, since translesional polymerase inactivity leads to higher susceptibility to DNA-toxic chemotherapy such as cisplatin [30,34]. Thus, when looking at the pathogenesis of MBS, REV3L has stood out as a strong candidate, and the neural defects encountered in mice align with the CNS lesions observed in MBS. Further research is required to ascertain whether different types of REV3L mutations are involved in a majority of MBS cases. However, it has yet to be explored whether other effects of these mutations (such as immunologic or carcinogenic) are present in MBS patients.

### 2.3. The Role of TUBB3 in Disease Pathogenesis in Human and Animal Models

**Figure 3:**
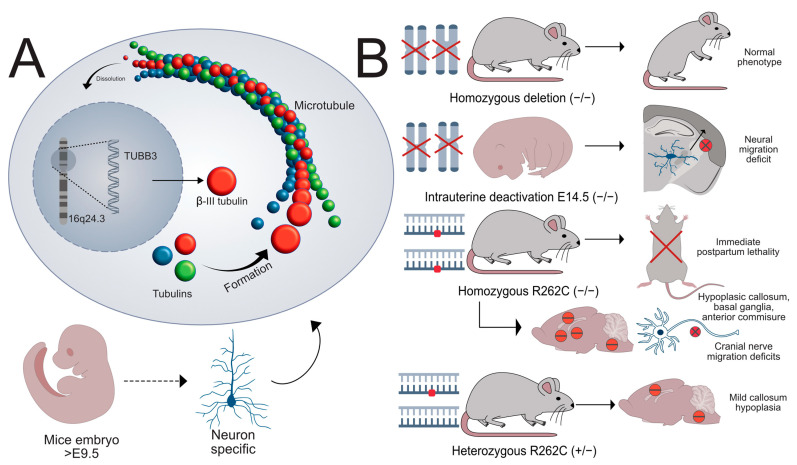
TUBB3 function: Image (**A**) details the role of the TUBB3 gene. Located on chromosome 16q24.3, it codes the protein β-III tubulin. Together with other members of the tubulin family, these proteins form microtubules. The process of microtubule formation and dissolution is continuous within cells. β-III tubulin appears in mice embryos starting from E9.5 and is neuron-specific, not being present in glial cells. Image (**B**) details the results of mice experiments into TUBB3 function. Homozygous deletion leads to normal phenotypes, with the other tubulins having compensatory expression. However, inactivation of the gene during the embryologic period (E14.5) leads to a breakdown of cortical neural migration. Homozygous R262C mutation (a mutation identified as disease-inducing for this gene) leads to lethality immediately after birth. These mice present multiple histological abnormalities such as hypoplasia of the corpus callosum, anterior commissure, and basal ganglia. They also have impaired migration of the cranial nerves, mostly affecting CN III, IV, and V. The heterozygous form of the R262C mutation produces only mild callosal hypoplasia.

TUBB3 (ID: 10381) is a gene found in chromosome 16q24.3 (NC_000016.10, GRCh38.p14) coding a member of the β-tubulin family of proteins. These proteins form the structure of microtubules within neurons (Figure 3A). As expected, the widest representation of this protein is in the CNS with broad distribution across other tissues. Testicular tissue also expresses TUBB3 in larger quantities [19]. TUBB3 is essential for neuronal development and is thought of as a highly specific marker for neurons, differentiating them from glial cels throughout the central and peripheral nervous systems (Figure 3A) [35,36,37]. However, expression becomes differentiated after the embryological phase, with TUBB3 maintaining higher expression levels in the peripheral than in the central nervous system [36]. Microtubular formation is a continuous process; as such, some level of TUBB3 expression is always maintained in mammalian tissues (Figure 3A). Embryologically, it is detectable in the neural crest even before migration in chickens, and it is present from the early phases of neural formation (9 dpc) in rats [38]. Within the brain, the posterior fossa structures have higher expressions of TUBB3 [38]. Mice knockout studies have revealed that total inactivation of TUBB3 (TUBB3 −/−) during the embryological phase does not produce noticeable pathological phenotypes, either in the general viability of such mice or in their main cortical structures (Figure 3B) [35]. Human studies have encountered altered brain structures such as the corpus callosum, anterior commissure, and basal ganglia in patients suffering from CFEOM with TUBB3 mutations (R262C, A302T, R62Q, R380C, R262H, E410K, D417N). These alterations lead to cognitive impairment when present [37]. The most common of these mutations, R262C, was also tested in mice in both heterozygous (wt/R262C) and homozygous (R262C/R262C) variants. It caused rapid postpartum lethality in homozygous (R262C/R262C) mice, with heterozygous mice (+/R262C) exhibiting normal phenotypes (Figure 3B). Histologically, +/R262C mice exhibited only a mildly hypoplastic anterior commissure, while R262C/R262C mice presented with abnormalities of the other commissural structures and the basal ganglia. Furthermore, oculomotor, trochlear, and trigeminal nerve growth defects or pathfinding abnormalities were encountered (Figure 3B) [39]. These are consistent with patients exhibiting the same mutation and CFEOM phenotype [38]. Embryological age seems to play a role in this variance in the response to TUBB3 alterations, as in the previous studies, no alteration to cortical development was observed [35,37], while an induced inactivation of TUBB3 in mice 14.5 dpc led to greatly impaired cortical migration of neurons (Figure 3B) [40]. Overall levels of tubulin proteins remained stable in whole-inactivation mice (−/−) and was slightly decreased in R262C/R262C mice [35,37]. However, it is clear that TUBB3 deficiency causes axonal migration impairment, affecting growth cone expansion rates [35]. This seems to be due to the increase in the stability of TUBB3-deficient microtubules, documented previously [37]. This, in turn, both affects axonal development during embryological phases and can slow down nerve repair post-injury, with the effect being observed in TUBB3 homozygous (−/−) loss-of-function mice and cell cultures [35]. These central axonal guidance defects in TUBB3 were shown to be related to a breakdown of netrin-1-induced neurite growth, explaining the slow repair rate post-injury or defective cranial nerve development [39].

In human disease, TUBB3 has primarily been quoted as responsible for congenital fibrosis of the extraocular muscles type 3 (CFEOM3) [5,17,18,19,41]. However, it has also been discussed as possibly involved in MBS, which is characterized by abduction palsy of the eyes [5]. CFEOM is a differential diagnosis for MBS, since it can be associated with other cranial nerve palsies. However, vertical gaze limitations exclude the diagnosis of classical MBS and are commonly found in CFEOM3, making the distinction between the two very difficult [5]. Furthermore, in a large cohort study, patients presenting with classical MBS (i.e., without vertical gaze palsy) had no mutations of the TUBB3 gene [5]. This pleads for the need for increased attention when clinically evaluating these patients, since they may be falsely classified as MBS or might meet the diagnostic criteria but suffer from a broader nervous system defect. Interestingly enough, an analysis of n = 454 reported cases of MBS found numerous cases where 3rd CN palsy was present (n = 45) or where blepharoptosis was present (n = 29) [1]. As such, it is our feeling that further discussion is needed as to the role of oculomotor deficits in MBS phenotypes. To complicate matters, a de novo mutation of TUBB3 (Arg262His) was encountered in a series of patients presenting with the minimum diagnostic criteria for MBS, with some being diagnosed clinically as such, but with important associated malformations. Of these, we mention growth retardation, vocal fold paralysis, facial dysmorphism, joint contracture, and CNS abnormalities such as hypotrophy of the corpus callosum and basal ganglia dysgenesis. The series can be classified as a new and severe association between CFEOM3 and MBS [17]. Milder variants of eye abnormalities have been reported for the M323V mutation, which leads only to congenital nystagmus [42]. TUBB3 acquired mutations have also been associated with different types of cancers such as prostate [43], pulmonary [44], renal [45], gallbladder [46], and breast [47]. TUBB3 mutations in these cases lead to an aggressive and chemo-resistant phenotype [43,44,45,46], and its knockdown prevented metastatic spread to the brain in both cell line and mouse experiments [47]. It is essential to further analyze TUBB3 mutations in MBS patients and associated animal models in order to properly define the interplay between this mutation and the different phenotypes related to the disease. It is also essential to further explore the interplay between this gene and the other main candidates for the genetic etiology of MBS.

### 2.4. Animal Models for Vascular Congenital Disorders of the Cranial Nerves

Lesions of the 6th and 7th nerve nuclei within the pons have been observed in numerous MBS patients across various series [1,2,6,14,48,49,50]. These have been reported in the form of hypoplasia, calcifications or necrotic lesions on MRI, and histological data from MBS patients [1,2,6,10,49,50]. Similar lesions are encountered in other cranial nerve palsies, which is why some authors have grouped these pathologies under the larger umbrella of “brainstem dysgenesis” [6]. Even for CFEOM 3, a pathology previously discussed in this article and others as highly linked to the TUBB3 mutation [5,38], similar findings are encountered on MRI scans of the CNS [13]. It was only natural, then, that in these cases a theory of congenital vascular defects would be considered the most plausible etiology. As such, even from the 1980s, studies have appeared suggesting a vascular etiology for cranial nerve-related congenital diseases [8,9], with most later studies supporting this theory [1,2,6,48,49].

**Figure 4:**
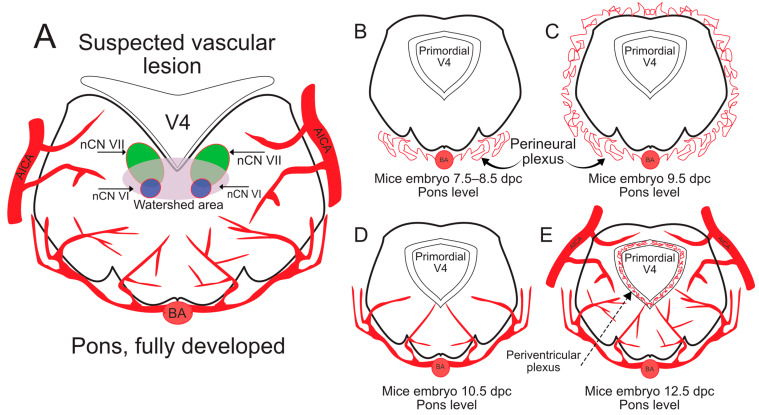
Vascular development and pathogenic theories in mice: Image (**A**) is a coronal section at the level of the pons in adult mice. The nuclei of the facial nerve (nCN VII) and of the abducens nerve (nCN VI) are located near the floor of the ventricle. The area is located posteriorly, distant from the main arteries, leading to the formation of a less densely vascularized area called the “watershed area”. Image (**B**) is a coronal section at the level of the pons between 7.5–8.5 dpc. We can observe the incipient development of an anterior vascular plexus. Image (**C**) is a coronal section at the level of the pons at 9.5 dpc. The vascular plexus has surrounded the entirety of the neural tube. Image (**D**) is a coronal section at the level of the pons at 10.5 dpc. The plexus is organized into blood vessels surrounding the neural tubes with ramifications forming and incipient penetration of the neural tube. Image (**E**) is a coronal section at the level of the pons at 12.5 dpc. The arteries have continued ramifying and have entered the neural tissue, leading to the formation of a periventricular plexus.

The vulnerability of cranial nerve nuclei of CN III, V, VI, and VII to vascular problems comes from their localization parasagittal on the floor of the 4th ventricle or within the posterior aspect of the mesencephalic tegmentum [51]. This area is considered to be a “watershed” zone (Figure 4A). Since the vascularization of the brainstem comes from circumflex arteries arising from the basilar trunk and its tributaries [49,51], the very midline and adjacent area receives the terminal-most vascular supply, consequently being prone to infarction.

The necessity for an embryonic model of brainstem vascular lesions is evident in the case of MBS, since, as previously described, no genetic defect fully explains the development of this disease. However, obtaining a working mouse model where the phenotype of MBS is present has remained an elusive goal. It is important to note that the cerebral vascular system in mice differs from that of humans in that the basilar system is not connected to the supratentorial system [52], with some mice species being prone to medulla and pons infarctions similar to Wallenberg’s infarctions in humans [52]. Angiogenesis for the CNS in mice starts around 7.5 to 8.5 dpc with formations of vascular plexuses on the ventral aspect of the neural tube (Figure 4B). By day 9.5, the larger branches have surrounded the neural tube and start migrating toward the developing ventricles (Figure 4C) [53]. Branching of the vessels becomes visible by 10.5 dpc (Figure 4D), with a complex plexus formed in the periventricular space visible by 12.5 dpc (Figure 4E) [54]. MR angiograms of the developing CNS vasculature has been described as a tool for studying mice embryos starting from 11.5 dpc, with the method becoming increasingly more accurate from 12.5 dpc [55]. The method has been proven to detect even microvascular lesioning in mutant Gli 2 knockout (−/−) mice, emerging as a useful tool to study the interaction between genetic defects and vascular abnormalities in mice [55].

Other promising vascular models for the CNS have been described. The first studies relating to cranial nerve defect syndromes observed lesions in the nuclear area of the brainstem, preferentially in the dorsal brainstem, during temporary systemic circulatory arrest in rhesus monkeys [50]. Another emerging model is that of the zebrafish, which allows for easy in vivo fluorescence imaging of the developing CNS [54]. While the projection of the facial nerve for zebrafish is significantly different from that of humans [56], the zebrafish model has been proven to be reliable in studying both the vascular system [54] and plexin-related axonal guidance deficits [56]. This makes it an ideal candidate for the study of the primary brainstem and axonal guidance deficits involved in MBS etiology. Chick embryos are also an easily accessible model for the study of CNS vasculature and were extensively used in the past [57,58]; this, combined with their use for neuronal pathfinding defects [59], would make them another candidate for the study of the interrelation between vascular and genetic defects in MBS.

## 3. Discussion

With MBS being such an elusive and rare pathology, it is usually very hard for clinicians unfamiliar with it to establish the diagnosis and refer patients to national or international specialized centers. This is probably why the incidence of MBS is still only an estimate [2,10]. The existence of a working model for replicating Moebius phenotypes within experimental animals would greatly improve our chances of determining the exact pathogenic process leading to this phenotype. All the main genetic and vascular factors discussed in the previous sections have mice models documented in the literature; however, none of these models exhibit MBS phenotypes, while the genetic findings in patients suffering from MBS are, at best, inconsistent [14,16]. This leads us to believe that development of the MBS phenotype might be caused by an association of these factors, combining local or systemic mutagenesis with vascular deficiencies exacerbated or induced by these mutations. Thus, we feel that it is necessary to study all these etiologies concomitantly within an animal model to have the possibility of expressing the MBS phenotype. The murine model would be the preferred vector for evaluating MBS development from an observational standpoint, since phenotypic evaluation can be performed through standardized tests. Furthermore, the relative ease with which transgenic variants can be obtained makes it the ideal vector for testing the genetic etiology of MBS. It is important to note that the studies detailing genetic mutations in the case of this disease [14,16] and studies discussing the possible etiology of the syndrome [10] have screened for and identified only single-nucleotide change mutations. Other types of mutations have not been explored, although some studies mention chromosomal defects [10]. While previous studies performed experiments on loss-of-function heterozygous or homozygous mice for the PLXND1 and REV3L genes [14], no study exists that tested the individual mutations highlighted in patients suffering from MBS. We consider it crucial to test individual mutations as described in the previous sections, since in the case of TUBB3, testing of the R262C single-nucleotide mutation resulted in a different phenotype than the loss-of-function mutation [39]. This is now more generally accessible due to the wider adoption of CRISPR-CAS genetic alteration tools, which allow for multiple single-nucleotide changes or even epigenetic inhibitory or activating mutations [60]. The same technology can be conceivably used to create a more targeted screening test for patients suffering from MBS instead of the current use of wide-genome non-specific tests [16,60]. It is important also to consider the chick embryo and zebrafish embryo models alongside the murine models, since the ease of access to developing neural structures can provide useful molecular insights into the underlying mechanisms of MBS pathogeny. The authors’ proposition for the vascular model would see an experimental lesioning of the posterior aspect of the pons in a chick embryo model under microscopic guidance. This model would allow for easy control of the lesioning process and permit inspection of the affected site during embryological development. The authors propose direct lesioning using a micro-needle or similar instruments; however, sclerotic agents could also be tested to better mimic the proposed natural form of occurrence. However, the use of sclerotic agent injections offers less control, with the possibility of wash-out of the agent or diffusion into unwanted vascular branches. The optimal moment for this injection would be at the moment when vessels start appearing on the dorsal part of the chick neural tube: Hamburger–Hamilton (HH) stage 24 or days 4–5 of development [61]. The development of the facial nerve, however, occurs during HH stage 17 to 28, with the formation of the geniculate ganglion starting around HH 23 [62]. Thus, any mechanical lesioning mechanism should be applied around this stage as well. Subsequent histological evaluation can be carried out at different stages to highlight any changes in the development of facial area muscles. Their role is, however, limited only to this, since in vivo observation of the phenotype in these models is not possible. Our aim is to continue this work toward the goal of obtaining a functional animal model for MBS, facilitating further research in this rare but severe pathology.

## 4. Materials and Methods

**Figure 5:**
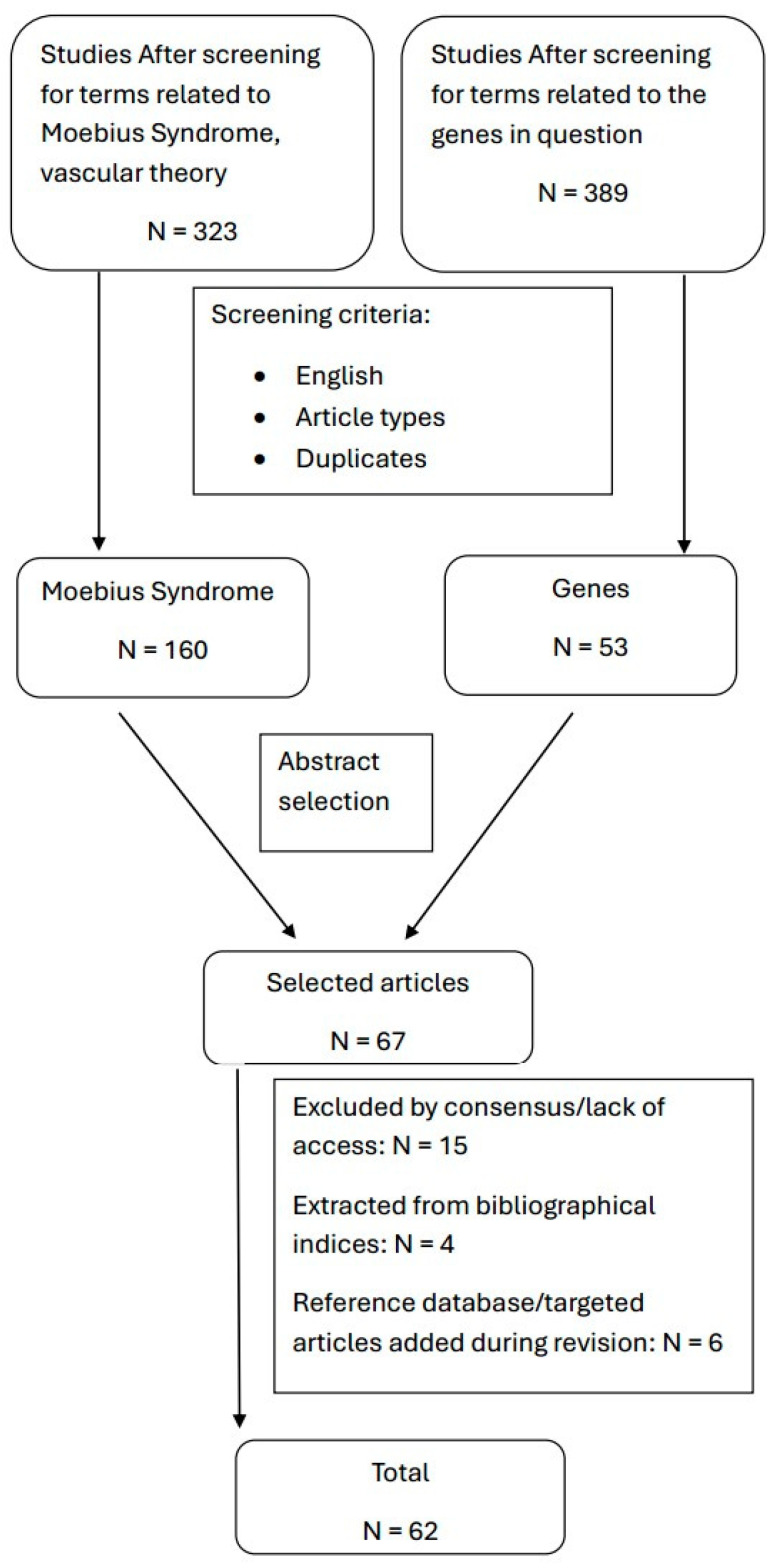
Flowchart detailing selection methodology for the review.

To perform this review, we searched the PUBMED database and Science Direct using combinations of the terms “Moebius syndrome”, “gene”, “genetic”, “etiology”, “vascular”, “mechanism”. We selected relevant review studies and case series regarding MBS in general and the vascular and genetic causes of MBS in particular. We identified the PLXND1, REV3L, and TUBB3 genes as possibly involved in the development of MBS. Thus, we searched the same databases for articles relating to the function of these genes and animal studies examining them. The terms used were “animal models”, “in mice”, “in mouse”, “in chicken embryo”, “zebrafish” in addition to the name of this gene. Our particular focus was on studies using mice. We also identified the possible vascular etiology of MBS and searched for articles detailing animal research into brainstem microvasculature. Terms used were “hindbrain vascular lesions”, “vasogenic involution of hindbrain”, “mouse cerebral vasculature”, “animal models”, “vascular embryogenesis”, “watershed area brainstem” (Figure 5). Included were original papers and review articles published in peer-reviewed international journals, reference books, and databases on the subjects discussed (Figure 5). We excluded articles not published in English, conference communications, dissertations, monographs, and other non-peer-reviewed forms of publication. After excluding duplicates, a total of 67 articles were selected for further analysis. Of these, 5 were excluded because of lack of access, and 10 were excluded by consensus. Four articles were extracted from bibliographic indices. To this, we added the reference “Gene” database and a number of 5 articles for the revision (Figure 5).

All figures were produced by the authors, using copyright-free vector images and personal drawings.

## Data Availability

The data presented in this study are available on request from the corresponding author (R.-V.T.).

## Abbreviations

The following abbreviations are used in this manuscript:

MBS	Moebius syndrome.
LOF	Loss of function.
DPC	Days post coitum.
